# Feasible logic Bell-state analysis with linear optics

**DOI:** 10.1038/srep20901

**Published:** 2016-02-15

**Authors:** Lan Zhou, Yu-Bo Sheng

**Affiliations:** 1 College of Mathematics & Physics, Nanjing University of Posts and Telecommunications, Nanjing, 210003, China; 2 Key Lab of Broadband Wireless Communication and Sensor Network Technology, Nanjing University of Posts and Telecommunications, Ministry of Education, Nanjing, 210003, China

## Abstract

We describe a feasible logic Bell-state analysis protocol by employing the logic entanglement to be the robust concatenated Greenberger-Horne-Zeilinger (C-GHZ) state. This protocol only uses polarization beam splitters and half-wave plates, which are available in current experimental technology. We can conveniently identify two of the logic Bell states. This protocol can be easily generalized to the arbitrary C-GHZ state analysis. We can also distinguish two *N*-logic-qubit C-GHZ states. As the previous theory and experiment both showed that the C-GHZ state has the robustness feature, this logic Bell-state analysis and C-GHZ state analysis may be essential for linear-optical quantum computation protocols whose building blocks are logic-qubit entangled state.

Quantum entanglement is of vice importance in future quantum communications, quantum computation and some other quantum information processing procotols^1^,^2^,^3^,^4^,^5^. For example, quantum teleportation^1^, quantum key distribution (QKD)^2^, quantum secret sharing (QSS)^3^, quantum secure direct communication (QSDC)^4^,^5^,^6^, quantum repeater^7^,^8^ and other important quantum information processing^9^,^10^,^11^,^12^,^13^,^14^,^15^,^16^ all require the entanglement. For an optical system, the photonic entanglement is usually encoded in the polarization degree of freedom. Besides the polarization entanglement, there are some other types of entanglement, such as the hybrid entanglement^17^,^18^,^19^,^20^,^21^, in which the entanglement is between different degrees of freedom of a photon pair. The photon pair can also entangle in more than one degree of freedom, which is called the hyperentanglement^22^,^23^,^24^,^25^,^26^,^27^,^28^,^29^. Both the hybrid entanglement and the hyperentanglement have been widely used in quantum information processing^30^,^31^,^32^,^33^,^34^,^35^.

Different from the entanglement encoded in the physical qubit directly, logic-qubit entanglement encodes the single physical quantum state which contains many physical qubits in a logic quantum qubit. Logic-qubit entanglement has been discussed in both theory and experiment. In 2011, Fröwis and Dür described a new kind of logic-qubit entanglement, which shows similar features as the Greenberger-Horne-Zeilinger (GHZ) state^36^. This logic-qubit entangled state is named the concatenated GHZ (C-GHZ) state. It is also called the macroscopic Schrödinger’s cat superposed state^37^,^38^,^39^,^40^,^41^,^42^,^43^. The C-GHZ state can be written as


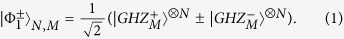


Here, *N* is the number of logic qubit and *M* is the number of physical qubit in each logic qubit, respectively. States 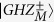 are the standard *M*-photon polarized GHZ states as


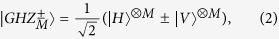


where 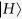 is the horizonal polarized photon and 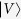 is the vertical polarized photon, respectively. Fröwis and Dür revealed that the C-GHZ state has its natural feature to immune to the noise^36^. Recently, He *et al.* demonstrated the first experiment to prepare the C-GHZ state^42^. In their experiment, they prepared a C-GHZ state with *M* = 2 and *N* = 3 in an optical system. They also investigated the robustness feature of C-GHZ state under different noisy models. Their experiment verified that the C-GHZ state can tolerate more bit-flip and phase shift noise than polarized GHZ state. It shows that the C-GHZ state is useful for large-scale fibre-based quantum networks and multipartite QKD schemes, such as QSS schemes and third-man quantum cryptography^42^.

On the other hand, similar to the importance of the controlled-not (CNOT) gate to the standard quantum computation model, Bell-state analysis plays the key role in the quantum communication. The main quantum communication branches such as quantum teleportation, QSDC all require the Bell-state analysis. The standard Bell-state analysis protocol, which utilizes linear optical elements and single-photon measurement can unambiguously discriminate two Bell-states among all four orthogonal Bell states^44^,^45^,^46^. By exploiting the ancillary states or hyperentanglement, four polarized Bell states can be improved or be completely distinguished^31^,^47^,^48^. For example, with the help of spatial modes entanglement, Walborn *et al.* described an important approach to realize the polarization Bell-state analysis^31^. The Bell-state analysis for hyperentanglement were also discussed^33^,^49^,^50^,^51^. By employing a logic qubit in GHZ state, Lee *et al.* described the Bell-state analysis for the logic-qubit entanglement^52^. The logic Bell-state analysis with the help of CNOT gate, cross-Kerr nonlinearity and photonic Faraday rotation were also described^53^,^54^,^55^. Such protocols which based on CNOT gate, cross-Kerr nonlinearity and photonic Faraday rotation are hard to realize in current experiment condition.

In this paper, we will propose a feasible protocol of logic Bell-state analysis, using only linear optical elements, such as polarization beam splitter (PBS) and half-wave plate (HWP). Analogy with the polarized Bell-state analysis, we can unambiguously distinguish two of the four logic Bell states. This approach can be easily generalized to the arbitrary C-GHZ state analysis. We can also identify two of the *N*-logic-qubit C-GHZ states. As the logic-qubit entanglement is more robust than the polarized GHZ state, this protocol may provide a competitive approach in future quantum information processing.

## Results

**Figure 1:**
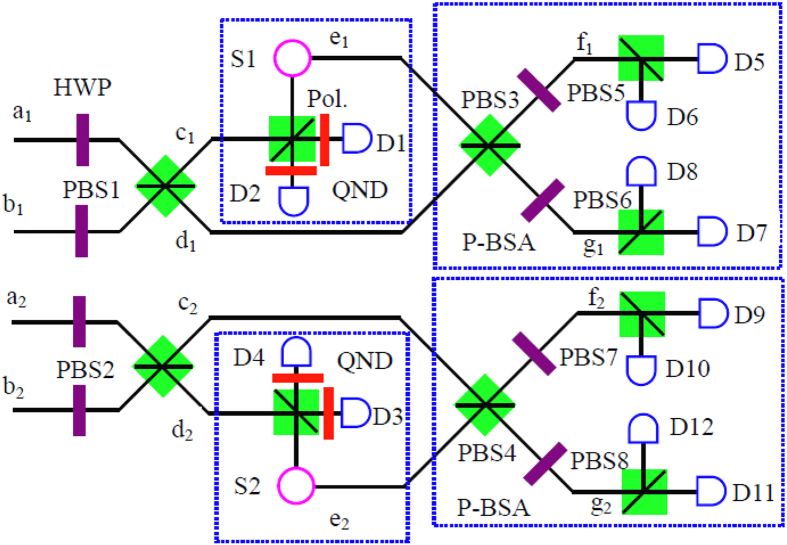
Protocol for logic Bell-state analysis. The QND is the teleportation-based probabilistic quantum nondemolition measurement with an ancillary entangled photon pair, which is first experimentally demonstrated in the hyperentanglement Bell-state analysis^34^. An incoming photon can cause a coincidence detection after the beam splitter. Subsequently, it can herald its presence and meanwhile can faithfully teleport its arbitrary unknown quantum state to a free-flying photon for further application. The P-BSA is the polarization Bell-state analysis, which can completely distinguish 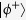 from 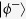. Pol. is the linear polarizer.

The basic principle of our protocol is shown in Fig. 1. The four logic Bell states can be described as


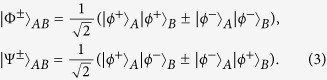


Here, 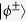 and 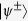 are four polarized Bell states of the form


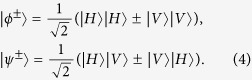


States in Eq. (3) can be regarded as the case of C-GHZ state in Eq. (1) with *N* = *M* = 2.

From Fig. 1, we first let four photons pass through four HWPs, respectively. The HWP can make 
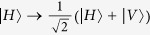
, and 
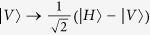
. The HWPs will make the state 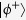 not change, while 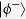 become 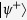. Therefore, after passing through four HWPs, the four logic Bell states can evolve to


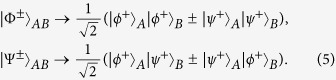


States 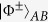 can be written as


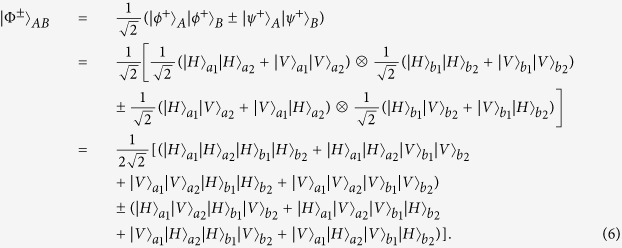


States 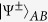 can be written as


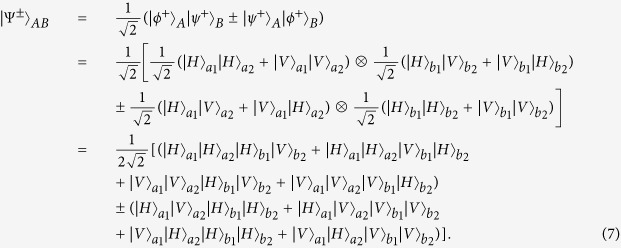


Subsequently, we let four photons pass through the PBS1 and PBS2, respectively. The PBS can fully transmit the 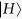 polarized photon and reflect the 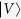 polarized photon, respectively. By selecting the cases where the spatial modes *c*_1_, *d*_1_, *c*_2_ and *d*_2_ all contain one photon, 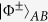 will collapse to


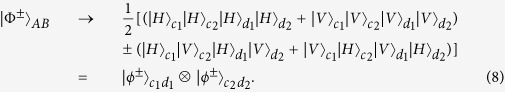


On the other hand, states 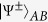 cannot make all the spatial modes *c*_1_, *d*_1_, *c*_2_ and *d*_2_ contain one photon. For example, item 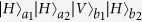 will make spatial mode *d*_1_ contain two photons but spatial mode *c*_1_ contain no photon. Item 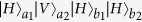 will make spatial mode *c*_2_ contain two photons, but no photon in the spatial mode *d*_2_.

In order to ensure all the four spatial modes contain one photon, our approach exploits quantum non-demolition (QND) measurement. It means that a single photon can be observed without being destroyed, and its quantum information can be kept. Quantum teleportation is a powerful approach to implement the QND measurement. Adopting the quantum teleportation to implement the QND measurement for realizing the Bell state analysis was first discussed in ref. 34. It will be detailed in Method Section.

After both successful teleportation, states 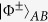 become 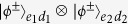, while states 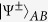 never lead to both successful teleportation. States 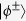 can be easily distinguished with polarization Bell-state analysis (P-BSA)^56^, as shown in Fig. 1. Briefly speaking, we let the four photons pass through two PBSs and four HWPs for a second time, respectively. After that, state 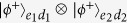 will not change, while state 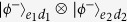 will become 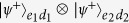. According to the coincidence measurement, we can finally distinguish the states 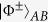. For example, if the coincidence measurement result is one of D5D7D9D11, D5D7D10D12, D6D8D9D11 or D6D8D10D12, the original state must be 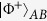. On the other hand, if the coincidence measurement result is one of D5D8D9D12, D5D8D10D11, D6D7D9D12 or D6D7D10D11, it must be 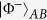. In this way, we can completely distinguish the states 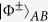.

In this protocol, each logic qubit is encoded in a polarized Bell state. Actually, if the logic qubit is encoded in a *M*-photon GHZ state, we can also discriminate two logic Bell states. The generalized four logic Bell states can be described as


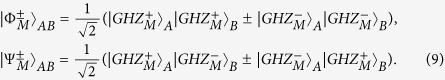


In order to explain this protocol clearly, we first let *M* = 3 for simple. If *M* = 3, the three-photon polarized GHZ states 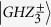 can be written as


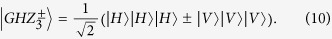


After performing the Hadamard operation on each photon, states 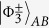 and 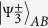 can be transformed to


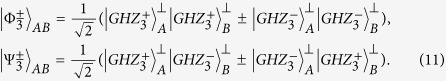


Here


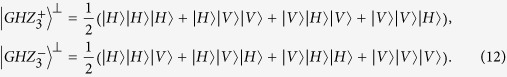


From Eq. (11), after performing the Hadamard operation, compared with the states in Eq. (9), states 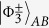 and 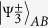 have the different form. The 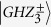 cannot be transformed to another GHZ state, which is quite different from the Bell states. States 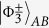 can be rewritten as


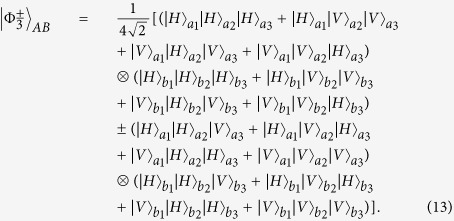


From Fig. 1, if the logic qubit is three-photon polarized GHZ state, we should add the same setup in spatial modes *a*_3_ and *b*_3_, as it is in *a*_1_ and *b*_1_. Certainly, we require three QNDs to complete the task. If we pick up the case that all the spatial modes *c*_1_, *d*_1_, *c*_2_, *d*_2_, *c*_3_ and *d*_3_ contain one photon, states 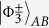 will collapse to


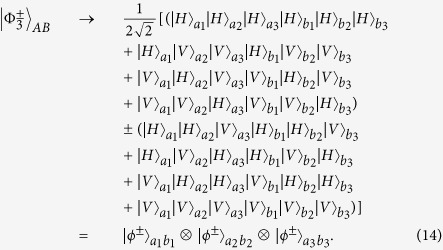


In order to complete such task, we require three pairs of polarized entangled states as auxiliary to perform the QND and coincidence measurement. States 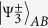 never lead to the case that all the spatial modes *c*_1_, *d*_1_, *c*_2_, *d*_2_, *c*_3_ and *d*_3_ contain one photon, which can be excluded automatically. The next step is also to distinguish the state 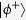 from 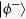, which is analogy with the previous description. In this way, we can completely distinguish the state 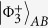 from 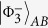.

Obviously, this approach can be extended to distinguish the logic Bell-state with the logic qubits encoded in the *M*-photon GHZ state 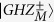, by adding the same setup in the spatial modes *a*_3_ and *b*_3_, *a*_4_ and *b*_4_, ···, and so on. With the help of QNDs and coincidence measurement, we can pick up the cases where all the spatial modes *c*_1_, *d*_1_, *c*_2_, *d*_2_, ···, *c*_*M*_ and *d*_*M*_ exactly contain one photon, which make the states 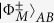 collapse to 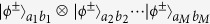. Each state 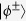 can be distinguished by the P-BSA. In this way, one can distinguish two logic Bell states with each logic qubit being the arbitrary *M*-photon GHZ state.

The GHZ state also plays an important role in fundamental tests of quantum mechanics and it exhibits a conflict with local realism for non-statistical predictions of quantum mechanics^57^. The first polarized GHZ state analysis was discussed by Pan and Zeilinger^56^. In their protocol, assisted with PBSs and HWPs, they can conveniently identify two of the three-particle GHZ states. Interestingly, our protocol described above can also be extended to the C-GHZ state analysis. The C-GHZ states can be described as


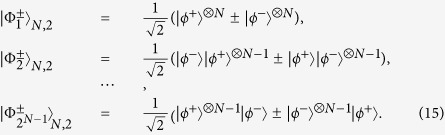


**Figure 2:**
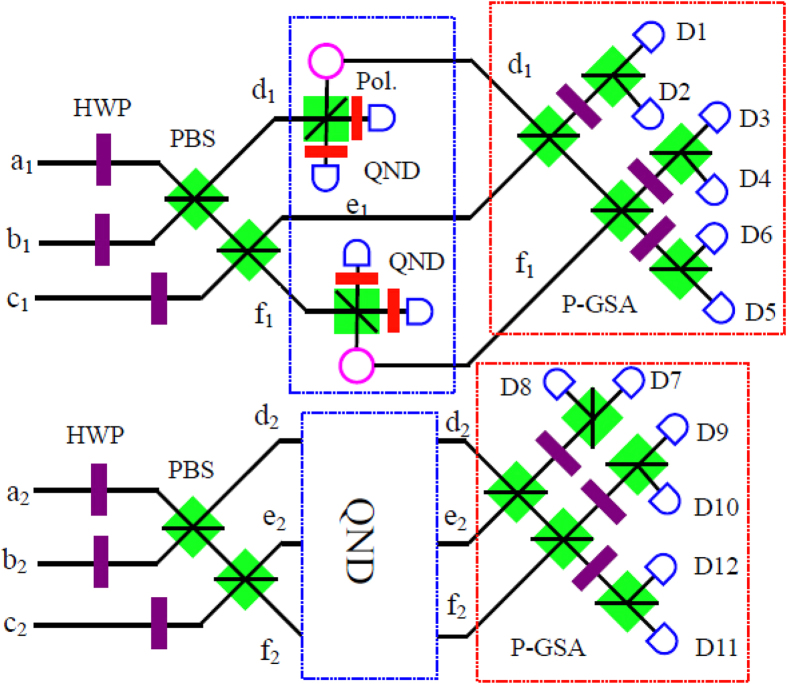
Protocol for C-GHZ state analysis with *N* = 3. The QND in the spatial modes *d*_2_, *e*_2_ and *f*_2_ is the same as the QND in *d*_1_, *e*_1_ and *f*_1_. The P-GSA is the polarized GHZ-state analyzer, which was first described in ref. 56.

We let the logic qubits be the Bell states 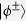 and still take *N* = 3 for example. From Fig. 2, after passing through the HWPs, the C-GHZ states can be described as


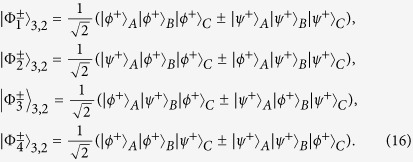


We let the six photons pass through four PBSs, respectively. If we pick up the cases in which all the spatial modes *d*_1_, *e*_1_, *f*_1_, *d*_2_, *e*_2_ and *f*_2_ exactly contain one photon, states 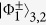 will become


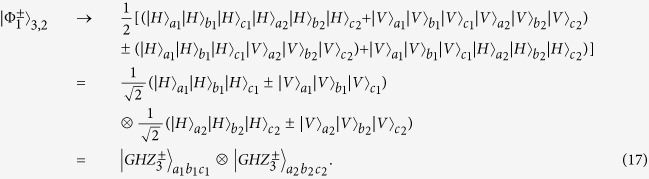


In order to complete this task, we also exploit the QNDs. As shown in Fig. 2, we require four QNDs, which are the same as those in Fig. 1. The QNDs in spatial modes *d*_2_, *e*_2_ and *f*_2_ are the same as those in the spatial modes *d*_1_, *e*_1_ and *f*_1_. From Eq. (17), if all the spatial modes *d*_1_, *e*_1_, *f*_1_, *d*_2_, *e*_2_ and *f*_2_ exactly contain one photon, the initial states 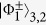 will collapse to the standard polarized GHZ states 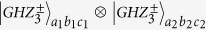. States 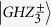 can be deterministically distinguished by the setup of polarized GHZ-state analysis (P-GSA), as shown in Fig. 2. The P-GSA was first described in ref. 56. Briefly speaking, 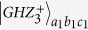 leads to coincidence between detectors D1D3D5, D1D4D6, D2D3D6 or D2D4D5, and 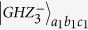 leads to coincidence between detectors D2D4D6, D1D4D5, D2D3D5 or D1D3D6. State 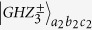 can be distinguished in the same principle. In this way, we can distinguish two states 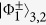 from the eight states as described in Eq. (16).

**Figure 3:**
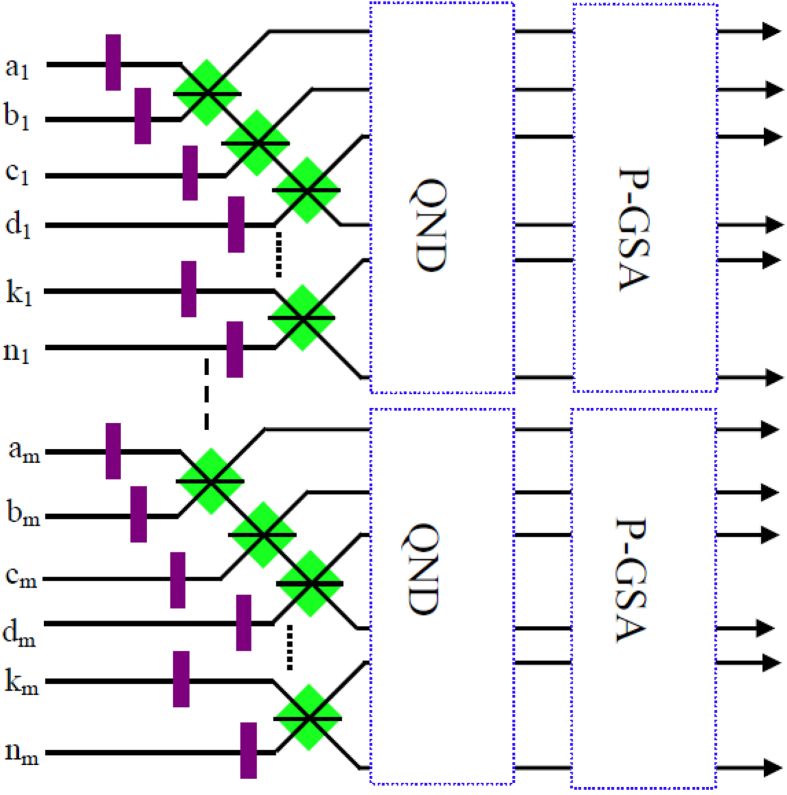
Protocol for C-GHZ state analysis with arbitrary *N* and *M*. The QNDs are used to ensure that each spatial mode contains one photon, which can project the original state to one of the *N*-photon polarized GHZ states 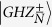. The P-GSA can distinguish 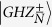
56.

For the *N*-logic qubit C-GHZ state analysis, this protocol can also work. As shown in Fig. 3, if each logic qubit is a Bell state, we let the photons in spatial modes *a*_1_, *b*_1_, ···, *n*_1_ and *a*_2_, *b*_2_, ···, *n*_2_ pass through the *N* − 1 PBS, respectively. By using QNDs to ensure each of the spatial modes behind the *N* − 1 PBSs contains one photon, it will project the states 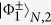 to 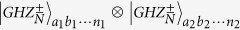, which can be completely distinguished by P-GSA as described in ref. 56. We can also distinguish two C-GHZ states with arbitrary *N* and *M*. By adding the same setup in the spatial modes *a*_3_, *b*_3_, ···, *n*_3_, ···, *a*_*m*_, *b*_*m*_, ···, *n*_*m*_, we can project the C-GHZ states to 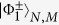 to 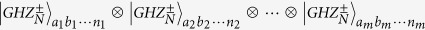, with the help of QNDs. Each pair of *N*-photon polarization GHZ states 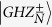 can be well distinguished. In this way, we can identify 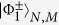 from arbitrary C-GHZ state completely.

## Discussion

So far, we have completely described our logic Bell-state and C-GHZ state analysis. In the logic Bell-state analysis, we can completely distinguish the states 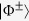 from the four logic Bell states. For arbitrary C-GHZ state analysis, we can also distinguish two states 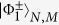 from the arbitrary *N*-logic-qubit C-GHZ states. It is interesting to discuss the possible experiment realization. In a practical experiment, one challenge comes from the multi-photon entanglement, for we require two polarization Bell states as auxiliary and the whole protocol requires eight photons totally. Fortunately, the eight-photon entanglement has been observed with cascaded entanglement sources^58^,^59^. The other challenge is the QND with linear optics^60^,^61^. From Fig. 2, the QND exploits Hong-Ou-Mandel interference^62^ between two undistinguishable photons with good spatial, time and spectral. As shown in ref. 34, the Hong-Ou Mandel interference of multiple independent photons has been well observed with the visibility is 0.73 ± 0.03. Different from ref. 34, we are required to prepare two independent pairs of entangled photons at the same time. This challenge can also be overcome with cascaded entanglement sources, which can synchronized generate two pairs of polarized entangled photons. This approach has also been realized in previous experimental quantum teleportation of a two-qubit composite system^63^. The final verification of the Bell-state analysis relies on the coincidence detection counts of the eight photons, with four photons coming from the QNDs and four coming from the P-BSA. This technical challenge of very low eight photon coincidence count rate was also overcome in the previous experiment by using brightness of entangled photons^58^,^59^. Finally, let us briefly discuss the total success probability of this protocol. In a practical experiment, we should both consider the efficiency of the entanglement source and single-photon detector. Usually, we exploit the spontaneous parametric down-conversion (SPDC) source to implement the entanglement source^64^. In order to distinguish C-GHZ state with *M* and *N*, we require (*M* − 1)*N* entanglement sources and [2 (*M* − 1) + *M*]*N* single-photon detectors. Suppose that the efficiency of the SPDC source is *p*_*s*_. A practical single-photon detector can be regarded as a perfect detection with a loss element in front of it. The probability of detecting a photon can then be given as *p*_*d*_. Therefor, the total success probability *P*_*t*_ can be written as


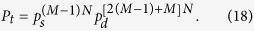


**Figure 4:**
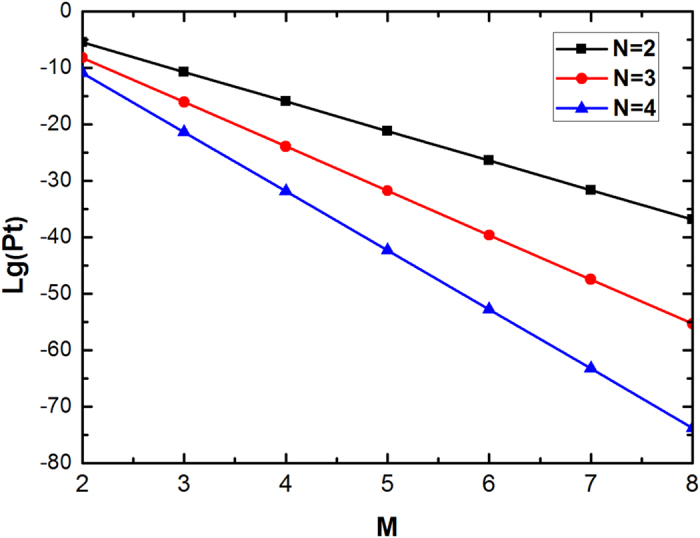
Schematic of the success probability altered with the physical qubit number *M*. Here we let *N* = 2, 3 and 4, respectively.

As point out in ref. 34, the mean numbers of photon pairs generated per pulse as *p*_*s*_ ~ 0.1. We let high-efficiency single-photon detectors with *p*_*d*_ = 0.9. We calculate the total success probability *P*_*t*_ altered with the *M* and *N*. If *M* = *N* = 2, we can obtain *P*_*t*_ ≈ 0.00656. In Fig. 4, the success probability is quite low, if *M* increases. From calculation, the imperfect entanglement source will greatly limit the total success probability. This problem can in principle be eliminated in future by various methods, such as deterministic entangled photons^65^.

In conclusion, we have proposed a feasible logic Bell-state analysis protocol. By exploiting the approach of teleportation-based QND, we can completely distinguish two logic Bell states 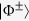 among four logic Bell-states. This protocol can also be extended to distinguish arbitrary C-GHZ state. We can also identify two C-GHZ states among 2^*N*^ C-GHZ states. The biggest advantage of this protocol is that it is based on the linear optics, so that it is feasible in current experimental technology. As the Bell-state analysis plays a key role in quantum communication, this protocol may provide an important application in large-scale fibre-based quantum networks and the quantum communication based on the logic qubit entanglement. Moreover, this protocol may also be useful for linear-optical quantum computation protocols whose building blocks are GHZ-type states.

## Methods

The QND is the key element in this protocol. Here we exploit the quantum teleportation to realize the QND. As shown in Fig. 1, both the entanglement sources *S*1 and *S*2 create a pair of polarized entangled state 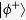, respectively. If the spatial mode *c*_1_ only contains a photon, a two-photon coincidence behind the PBS can occur with 50% success probability to trigger a Bell-state analysis. Meanwhile, both single-photon detectors *D*1 and *D*2 register a photon also means that we can identify 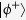 with the success probability of 1/4, which is a successful teleportation. It can teleport the incoming photon in the spatial mode *c*_1_ to a freely propagating photon in the spatial mode *e*_1_. On the other hand, if the spatial mode *c*_1_ contains no photon, the two-photon coincidence behind the PBS cannot occur. We can notice the case and ignore the outgoing photon. Using a QND in one of the arms of the PBS is sufficient. That is because the conserved total number of eventually registered photons for the case of two photon in spatial mode *c*_1_ or *d*_2_ can be eliminated automatically by the final coincidence measurement. In our protocol, the setup of teleportation can only distinguish one Bell state among the four with the success probability of the QND being 1/4. In this way, the total success probability of this protocol is 1/4 × 1/4 × 1/2 = 1/32. By introducing a more complicated setup of teleportation which can distinguish two polarized Bell states among the four^45^, the success probability can be improved to 1/2 × 1/2 × 1/2 = 1/8 in principle.

## Additional Information

**How to cite this article**: Zhou, L. and Sheng, Y.-B. Feasible logic Bell-state analysis with linear optics. *Sci. Rep.* **6**, 20901; doi: 10.1038/srep20901 (2016).
